# P-1594. Incidence and Severity of Post-Pandemic SARS-CoV-2 Infection during Pregnancy

**DOI:** 10.1093/ofid/ofaf695.1773

**Published:** 2026-01-11

**Authors:** Annette Regan, Matthew Coates, Sheena G Sullivan, Stacey Rowe, Flor M Munoz, Onyebuchi Arah

**Affiliations:** Kaiser Permanente Southern California, Pasadena, CA; University of California Los Angeles, Los Angeles, California; Monash University, Melbourne, Victoria, Australia; Kaiser Permanente Southern California, Pasadena, CA; Baylor College of Medicine Houston, Dallas, Texas; University of California Los Angeles, Los Angeles, California

## Abstract

**Background:**

Current vaccine policies prioritize pregnant people as a risk group for severe COVID-19. However, data supporting pregnancy as a risk factor for severe COVID-19 have been predominantly sourced during the Delta and early Omicron waves of the COVID-19 pandemic. Post-pandemic data on the health effects of SARS-CoV-2 during pregnancy are needed to ensure immunization and treatment policies remain based on current evidence.

**Figure:**
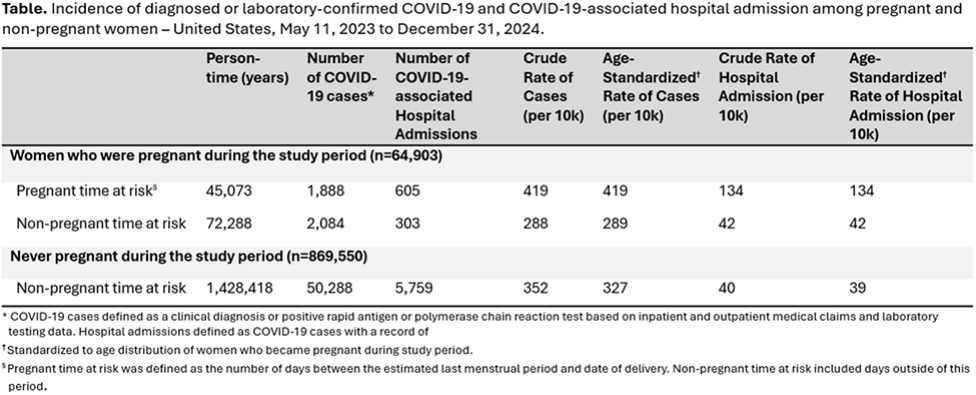

**Methods:**

We used de-identified commercial insurance claims from the Optum Labs Data Warehouse, a national de-identified administrative health care database including inpatient and outpatient medical claims, pharmacy records, laboratory testing records, and enrollment records for commercial and Medicare Advantage enrollees. Between May 11, 2023 (i.e., end of U.S. Public Health Emergency) to December 31, 2024, we identified a cohort of women aged 18 to 49 years. Pregnancy status was identified using a validated algorithm. We identified COVID-19 cases using ICD-10-coded inpatient and outpatient records and positive rapid antigen and polymerase chain reaction tests. We calculated crude and age-standardized rates of COVID-19 and COVID-19-associated hospitalizations per 10,000 person-years during pregnancy and outside of pregnancy.

**Results:**

Among 64,903 pregnant and 869,550 non-pregnant women, the age-standardized incidence of COVID-19 was 419/10,000 person-years during pregnancy and 327/10,000 person-years outside of pregnancy (Table 1); 32% (n=605/1,888) of COVID-19 cases in pregnancy resulted in hospitalization, and 11% (n=5,759/50,288) of cases outside of pregnancy resulted in hospitalization. The age-standardized incidence of COVID-19-associated hospitalization was 134/10,000 person-years in pregnancy and 39/10,000 person-years outside of pregnancy.

**Conclusion:**

Pregnancy did not appear to increase the risk of clinical detection of COVID-19, but did increase the risk of hospitalization among cases. While the incidence of COVID-19 was similar among pregnant and non-pregnant women, the incidence of hospitalization was 3.4-fold higher during pregnancy. During a period of endemic SARS-CoV-2 circulation, pregnant women continue to be at higher risk of severe infection requiring hospitalization.

**Disclosures:**

Annette Regan, PhD, MPH, Moderna: DSMB Membership Sheena G. Sullivan, PhD, Astra-Zeneca: Advisor/Consultant|CSL Behring and CSL Seqirus: Advisor/Consultant|GSK: Advisor/Consultant|Moderna: Advisor/Consultant|Novavax: Advisor/Consultant|Pfizer: Advisor/Consultant|Sanofi: Advisor/Consultant Stacey Rowe, PhD, MPH, CSL Seqirus: Advisor/Consultant Flor M. Munoz, MD, Merck: Advisor/Consultant|Pfizer: Advisor/Consultant|Pfizer: Grant/Research Support

